# Expression of PD-L1 on Monocytes Is a Novel Predictor of Prognosis in Natural Killer/T-Cell Lymphoma

**DOI:** 10.3389/fonc.2020.01360

**Published:** 2020-08-06

**Authors:** Xue-wen Zhang, Xi-wen Bi, Pan-pan Liu, Ze-long Liu, Man Nie, Hang Yang, De-xin Lei, Yi Xia, Wen-qi Jiang, Wei-an Zeng

**Affiliations:** ^1^State Key Laboratory of Oncology in South China, Collaborative Innovation Center for Cancer Medicine, Guangzhou, China; ^2^Department of Anesthesiology, Sun Yat-sen University Cancer Center, Guangzhou, China; ^3^Department of Medical Oncology, Sun Yat-sen University Cancer Center, Guangzhou, China; ^4^Department of Interventional Ultrasound, The First Affiliated Hospital of Sun Yat-sen University, Guangzhou, China

**Keywords:** natural killer/T-cell lymphoma, programmed death-ligand 1, monocyte, prognosis, immune therapy, tumor microenvironment

## Abstract

**Background:** Natural killer/T-cell lymphoma (NKTCL) is a highly aggressive lymphoma with a dismal prognosis, and novel therapeutic targets are urgently needed. Programmed death-ligand 1 (PD-L1) has become a promising therapeutic target for various cancers, but most of the studies have focused on expression of PD-L1 on tumor cells. Expression of PD-L1 on tumor-infiltrating non-malignant cells, especially monocytes, has not been studied in NKTCL, and its prognostic value remains unknown.

**Materials and Methods:** Expression of PD-L1 on tumor-infiltrating stromal cells was measured in NKTert and HS5 cells when cultured alone or co-cultured with NKTCL cell lines. Clinical samples were collected from 42 patients with newly diagnosed NKTCL. Expression of PD-L1 on monocytes was analyzed in patients' peripheral blood and tumor tissues using flow cytometry and immunofluorescent staining, respectively. Survival data were retrospectively collected and the prognostic significance of PD-L1 expression on monocytes was analyzed.

**Results:** PD-L1 expression on tumor-infiltrating stromal cells was remarkably elevated when co-cultured with NKTCL cells. The percentage of PD-L1+ monocytes among all monocytes in peripheral blood was significantly higher in NKTCL patients than that in healthy individuals. Among NKTCL patients, percentage of PD-L1+ monocytes in blood positively correlated with that in tumor tissues. Patients with a higher percentage (≥78.2%) of PD-L1+ monocytes in blood or with a higher percentage (≥24.2%) of PD-L1+ monocytes in tumor tissues exhibited a significantly inferior survival, compared with their counterparts. A higher percentage of PD-L1+ monocytes in blood or tumor tissues was an independent adverse prognostic factor.

**Conclusions:** Expression of PD-L1 on monocytes is up-regulated and has significant prognostic value in patients with NKTCL.

## Introduction

Natural killer/T-cell lymphoma (NKTCL) is a distinct subtype of non-Hodgkin lymphoma in World Health Organization (WHO) classification of hematopoietic and lymphoid malignancies (1–3). This disease exhibits highly aggressive histological and clinical features with a dismal prognosis (4, 5). Although novel regimens containing asparaginase have improved patients' outcome compared with anthracycline-based chemotherapy, novel therapeutic targets and risk-stratification parameters are urgently needed (6–10).

Immune therapy has become an important milestone for cancer treatment. Programmed cell death receptor-1 (PD-1) and ligand (PD-L1) are the most widely used targets for immune therapy in solid and hematopoietic malignancies (11–15). However, most of previous studies have focused on expressions of PD-1 and PD-L1 on tumor cells and tumor-infiltrating lymphocytes (TILs), which serve as both prognostic factors and therapeutic biomarkers (16, 17). In our previous study, PD-L1 has been up-regulated and correlates with poor prognosis in NKTCL cells, suggesting a role of immune escape in tumor genesis and progression of NKTCL (18). Additionally, anti-PD-1 antibody has yielded promising results in relapsed/refractory NKTCL, but accurate predictors for treatment efficacy is yet to be investigated (19, 20).

Tumor microenvironment (TME) plays a critical role in tumor genesis and development (21–23). Tumor infiltrating stromal cells take the major occupancy in TME (23–25), and monocytes take the maximum popularity in stromal cells (26–29). In previously studies, monocytes have enhanced proliferation as well as LMP1 expression of NKTCL cells, but their role in immune escape of NKTCL remains unexplored (29). Our present study is to explore the expression PD-L1 on monocytes, a major element of tumor-infiltrating stromal cells, and to examine its prognostic significance in patients with NKTCL. Our goal is to find a novel prognostic predictor and indicator to select NKTCL patients who are mostly possible to benefit from immune treatment.

## Materials and Methods

### Cell Lines and Co-culture System

NKTert and HS5 were human bone marrow stromal cell lines obtained from Professor Peng Huang's lab, and cultured as previously described (30). SNK-6 and NKYS were human NKTCL cell lines obtained from Professor Liang Wang's lab, and cultured as previously described (18, 31). All the cell lines mentioned above had been authenticated by DNA (STR) profiling.

NKTert and HS5 were first plated in 6-well cell culture plates and incubated for 12 h, allowing adherence. Then NKTert and HS5 were monocultured as the control groups or co-cultured with SNK-6 and NKYS at the ratio of 3:1 for 24 h as the experimental groups (29). The culture medium of SNK-6 and NKYS cells were also added in the control group, respectively. A cell-to-cell contact co-culture system was built as a model of NKTCL tumor microenvironment. After co-incubation, the suspended NKTCL cells (SNK-6 and NKYS) were removed and the adherent stromal cells (NKTert and HS5) were collected for further analysis.

### Western Blot Analysis

Cells were collected and washed with ice-cold phosphate-buffered saline (PBS). Then cells were lysed in RIPA lysis buffer (CWBiotech, Beijing, China) containing 1% phosphatase inhibitor and protease inhibitor (CWBiotech, Beijing, China) for 30 min on ice. After centrifugation at 15,000 g for 15 min at 4°C, the protein supernatant was collected and analyzed using the PierceTM BCA Protein Assay Kit (Thermo Scientific, USA). Same amount protein extractions (20 μg per lane) were applied by 12% sodium dodecyl sulfate polyacrylamide gel electrophoresis (SDS-PAGE) and transferred to poly vinylidene fluoride (PVDF) membranes (Millipore, Billerica, MA, USA). Membranes were exposed to primary antibodies against PD-L1 and GAPDH (1:1,000 dilution, CST, USA) at 4°C overnight, followed by exposure to respective secondary anti-rabbit and anti-mouse IgG antibodies (1:2,000 dilution, CST, USA) for 1 h at room temperature. Signals for labeled proteins were detected with LumiGLO reagent and Peroxide (CST, USA) and ChemiDoc Touch Imaging System (BIO-RAD, USA).

### Quantitative Real-Time Polymerase Chain Reaction (qRT-PCR) Analysis

qRT-PCR analysis was performed to quantify RNA expression of PD-L1. RNA level of GAPDH was measured and used as the internal control. Total RNA of NKTert and HS5 cells from control and experimental groups was extracted using Trizol reagent (Invitrogen, USA). One microgram of the total RNA was reversely transcribed into cDNA using Bestar™ qPCR RT Kit (DBI Bioscience, China). The qRT-PCR reaction was prepared in a total volume of 20 μl containing 10 μl DBI Bestar® SybrGreen qPCR Master Mix (DBI Bioscience, China), cDNA derived from 0.2 μg of input RNA, 5 pM each primer, and 7 μl double-distilled H_2_O. The PCR was run on Stratagene Mx3000P Real-Time PCR system (Agilent Technologies, USA). The fluorescent quantity PCR conditions were as follows: pre-denaturation at 95°C for 2 min, followed by 40 cycles of 94°C for 20 s, 58°C for 20 s, and 72°C for 30 s. Human PD-L1 forward primer sequence was: 5′-GGAGCCATCTTATTATGCCTT-3′, its reverse primer sequence was: 5′-TCACTTTGCTTCTTTGAGTTTGT-3′. Human GAPDH forward primer sequence was: 5′-TGTTCGTCATGGGTGTGAAC-3′, its reverse primer sequence was: 5′-ATGGCATGGACTGTGGTCAT-3′.

### Flow Cytometry Analysis

PD-L1 expression on the cell surface was analyzed by flow cytometry. Cells were collected and fixed with 4% formaldehyde for 15 min at room temperature. The samples were incubated with primary antibody against PD-L1 (1:400 dilution, CST, USA) for 1 h at room temperature, then washed with PBS and incubated with secondary FITC-conjugated rabbit IgG (1:100, eBioscience, San Diego, CA) for 30 min at room temperature. The samples were analyzed by CytoFLEX flow cytometry (Beckman Counter, CA, USA) according to the manufacturer's instructions. The level of PD-L1 on monocytes was quantified by determining the percentage of PD-L1 positive monocytes among all monocytes in PBMCs.

### Patients and Treatment

Forty-two patients who were newly-diagnosed with NKTCL between 2017 and 2019 according to the WHO classification of hematopoietic and lymphoid tumors were enrolled in our study (3). Blood samples, formalin fixed paraffin-embedded (FFPE) specimens, and written informed consent were obtained from all the patients at their first visit. The study protocol was approved by the ethics committee of Sun Yat-sen University Cancer Center and complied with country-specific regulatory requirements. The study was conducted in accordance with the Declaration of Helsinki and Good Clinical Practice guidelines. The clinical characteristics are summarized in Table 1. Twenty-nine (69.1%) patients received induction chemotherapy followed by radiotherapy (RT) as their primary treatment. Twelve (28.6%) patients received chemotherapy alone and one (2.4%) received chemotherapy followed by autologous stem cell transplantation. First-line chemotherapeutic regimens included AspaMetDex (6 patients, 14.3%), P-Gemox (32 patients, 76.2%), and other regimens (4 patients, 9.5%). The median follow-up period for this cohort was 9.1 (1.4–16.9) months.

**Table 1:** The clinical characteristics of patients with NK/T-cell lymphoma.

**Parameters (No., %)**	**PD-L1+ monocytes in blood [%, median (range)]**	***P-*value**	**PD-L1+ monocytes in tumor [%, median (range)]**	***P-*value**
Overall (42, 100%)	44.9 (1.0–98.8)		13.3 (0–56.3)	
Gender				
Male (29, 69%)	39.4 (1.0–98.8)	0.362	11.7 (0–47.1)	0.355
Female (13, 31%)	66.1 (5.7–94.1)		13.6 (1.3–56.3)	
Age				
< 60 (28, 67%)	33.5 (1.0–94.7)	0.109	14.3 (0–55.7)	0.660
≥ 60 (14, 33%)	62.2 (6.0–98.8)		12.2 (0–56.3)	
ECOG score				
0–1 (37, 88%)	39.4 (1.0–98.8)	0.026	11.7 (0–55.7)	0.001
≥ 2 (5, 12%)	81.8 (48.1–94.7)		43.6 (29.6–56.3)	
Ann Arbor stage				
I–II (35, 83%)	34.4 (1.0–98.8)	0.007	11.1 (0–55.7)	< 0.001
III–IV (7, 17%)	81.8 (48.1–94.7)		39.1 (29.6–56.3)	
B symptoms				
No (29, 69%)	59.3 (2.4–98.8)	0.111	16.6 (0–56.3)	0.111
Yes (13, 31%)	23.3 (1.0–90.7)		7.5 (0–27.0)	
LDH				
Normal (33, 79%)	34.3 (1.0–98.8)	0.471	13.6 (0–56.3)	0.890
Elevated (9, 21%)	46.4 (18.9–94.7)		11.1 (1.5–49.8)	
NKPI score				
0–1 (30, 71%)	31.8 (1.0–94.6)	0.002	9.3 (0–47.1)	0.002
2–4 (12, 29%)	81.7 (12.9–98.8)		34.6 (0–56.3)	
Treatment response				
CR (22, 52%)	36.9 (2.8–94.7)	0.420	12.5 (0–49.8)	0.830
non-CR (20, 48%)	52.1 (1.0–98.8)		13.5 (0–56.3)	
Disease progression				
No (31, 74%)	23.4 (1.0–94.7)	< 0.001	6.7 (0–38.7)	< 0.001
Yes (11, 26%)	81.8 (34.4–98.8)		39.1 (13.3–56.3)	

*CR, complete remission; ECOG, Eastern Cooperative Oncology Group; LDH, lactate dehydrogenase; NKPI, natural killer/T-cell lymphoma prognostic index*.

### Isolation of Monocytes in PBMCs From Peripheral Blood

Peripheral blood mononuclear cells (PBMCs) were isolated from NKTCL patients and healthy adult donors' peripheral blood using Lymphoprep density gradient separation (Axis-Shield, Norway) according to the manufacturer's instructions. The monocytes in PBMCs were classified and isolated using flow cytometry analysis (29).

### Immunofluorescence Analysis of PD-L1 and CD14 Expression

The two adherent human stromal cell lines (NKTert and HS5) from monoculture or co-culture system were performed by immunofluorescent staining to make a direct view of PD-L1 expression under different treatments. An anti-PD-L1 antibody conjugated with AlexaFluor 488 was applied (1:50 dilution, CST, USA). Pictures of PD-L1 expression status were examined and captured using a laser confocal microscope (OLYMPUS FV1000) under high magnification (1,000 × magnification).

Formalin fixed paraffin-embedded (FFPE) specimens for pathological diagnosis were collected from the same NKTCL patients described above. Four micrometer thick sections were cut and immunofluorescent staining was performed using a mouse monoclonal primary antibody against PD-L1 (1:100 dilution, R&D Systems, USA) and a rabbit monoclonal primary antibody against CD14 (1:100 dilution, Novus Biologicals, USA), which was used to mark monocytes (27). A secondary antibody conjugated with AlexaFluor 647 against mouse and a secondary AlexaFluor 488-conjugated antibody against rabbit (1:200, Absin Bioscience Inc, Shanghai, China) were also applied. DAPI (4′, 6-diamino-2-phenyindole) fluorochrome was used for nuclei staining of all cells.

PD-L1/CD14 double staining cells were quantified by determining the percentage of cells with membrane positively stained both by AlexaFluor 647 (red color) and AlexaFluor 488 (green color) among all monocytes under high magnification (400 × magnification) in an inverted fluorescence microscope (Leica DMIRB, Heidelberg, Germany). The cell counting was calculated manually under high magnification (400 × magnification) using Image Pro Plus 6.0 software (Media Cybernetics, Maryland, USA) (18).

### Statistical Analysis

Each experiment was replicated at least three times. Comparisons between groups were performed using the student's *t*-test. Receiver operating characteristic (ROC) analysis was used to determine the accuracy of PD-L1 expression on monocytes in predicting disease progression. The point with the maximum Youden index in the ROC curve, defined as (sensitivity + specificity) −1, was chosen as the cut-off value for PD-L1 percentage. Correlations between clinical parameters and PD-L1 expression on monocytes were assessed using the Mann-Whitney *U*-test. Progression free survival (PFS) and overall survival (OS) were calculated by the Kaplan–Meier method and compared using the log-rank test. The prognostic factors were examined by univariate analysis. Variables with statistical significance in univariate analysis were included in the multivariate analysis using a stepwise forward Cox regression model. Differences were considered statistically significant at a two-sided *P* < 0.05. The statistical analysis was performed using SPSS version 17.0 (SPSS Inc., Chicago, IL, USA).

## Results

### PD-L1 Expression on Stromal Cells Was Up-Regulated by NKTCL Cell Lines

**Figure 1:**
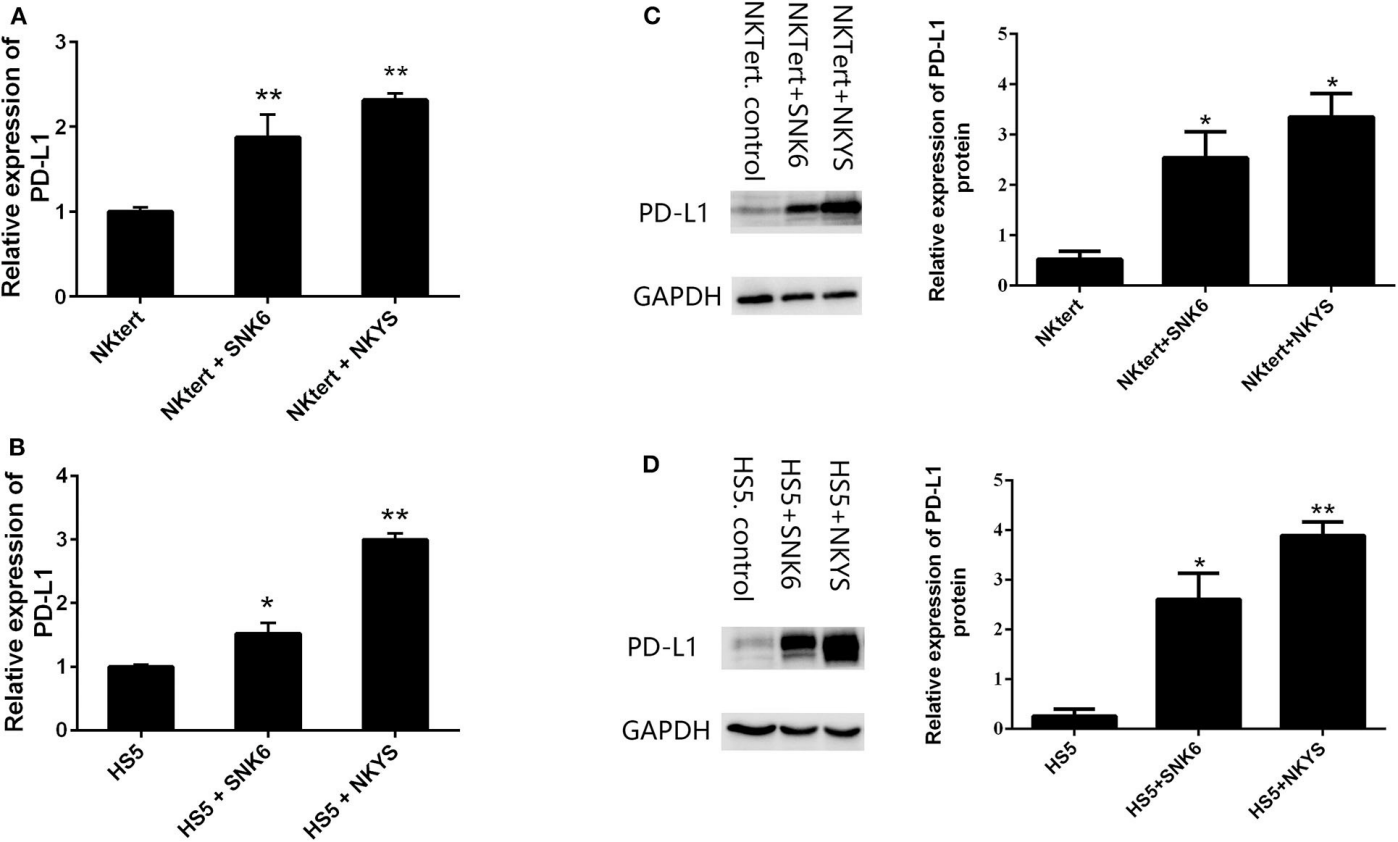
Expression of PD-L1 on human bone marrow stromal cell lines. Stromal cells (NKTert and HS5) were monocultured or co-cultured with NKTCL cells (SNK-6 and NKYS). **(A,B)** The level of PD-L1 mRNA was detected by quantitative real-time PCR (*n* = 3), *P < 0.01, **P < 0.001 by student's *t*-test. **(C,D)** The level of PD-L1 protein was detected by western blot (*n* = 3), *P < 0.05, **P < 0.01 by student's *t*-test.

**Figure 2:**
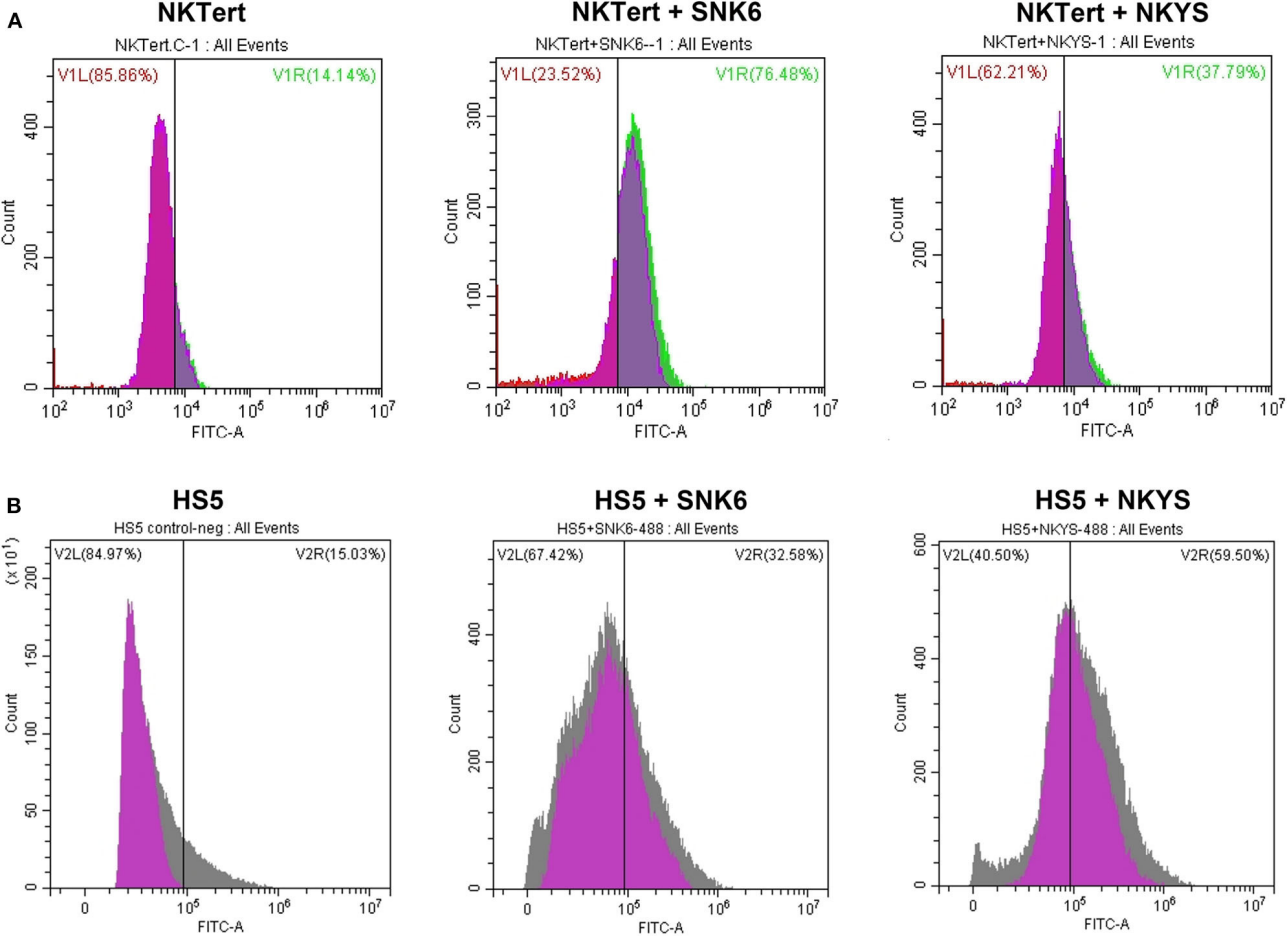
Expression of PD-L1 on human bone marrow stromal cell lines detected by flow cytometry. The percentage of PD-L1+ **(A)** NKTert and **(B)** HS5 cells were examined when monocultured or co-cultured with NKTCL cells (SNK-6 and NKYS).

**Figure 3:**
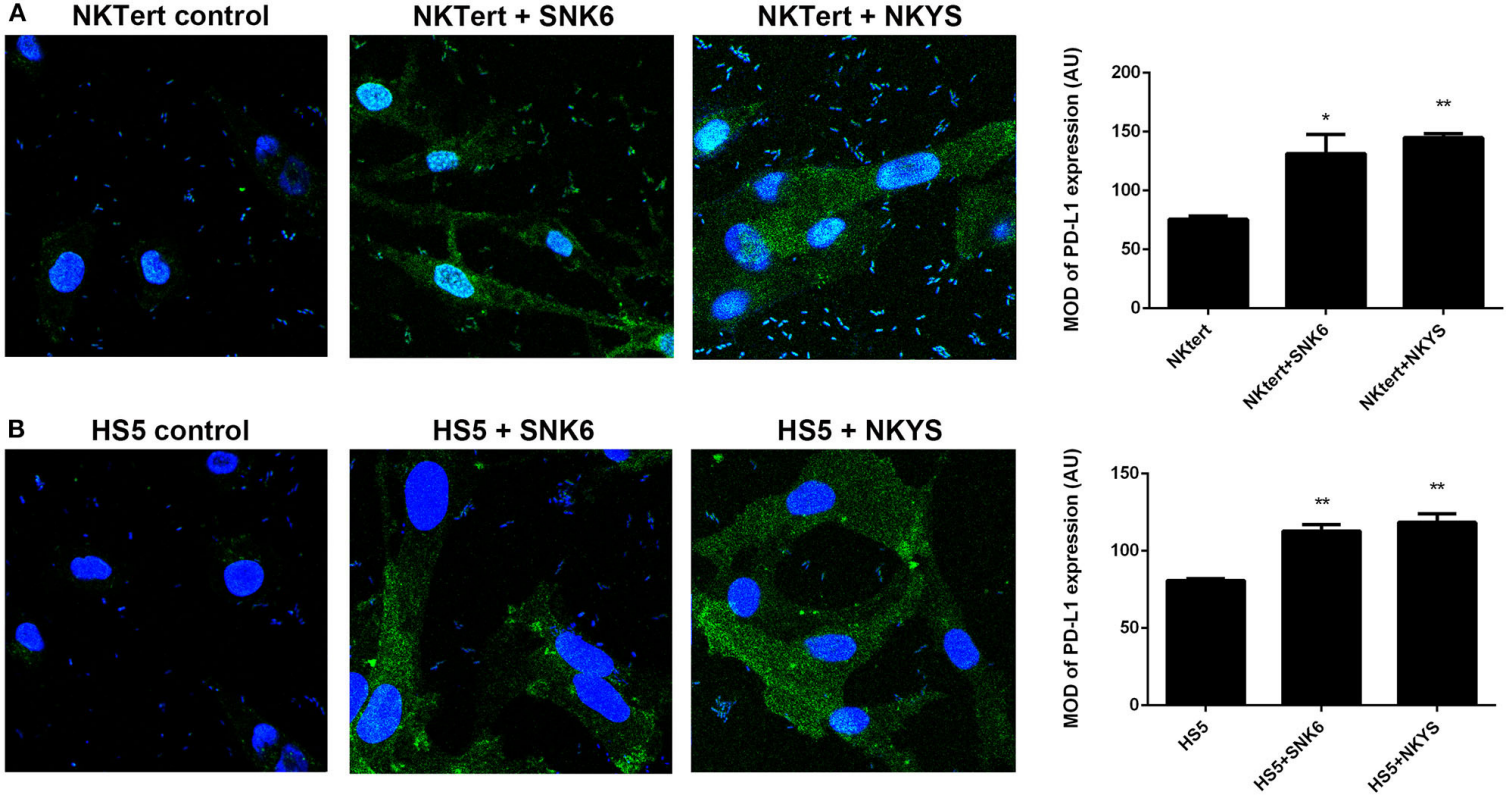
Expression of PD-L1 on human bone marrow stromal cell lines detected by immunofluorescence. Immunofluorescent staining of PD-L1 (green color) on **(A)** NKTert and **(B)** HS5 cells when monocultured or co-cultured with NKTCL cells (SNK-6 and NKYS), 600 × magnification, *n* = 3, *P < 0.05, **P < 0.01 by student's *t*-test, MOD, mean optical density; IOD, integrated optical density; AU, Arbitrary Units; MOD, IOD/Area.

PD-L1 expression was examined in human bone marrow stromal cells (NKTert and HS5) either when they were cultured alone or co-cultured with NKTCL cells (SNK-6 and NKYS). qRT-PCR and western blot revealed remarkably elevated levels of PD-L1 mRNA and protein in stromal cells, respectively, when co-cultured with NKTCL cells (Figures 1A–D). Consistently, flow cytometric and immunofluorescent analysis showed higher levels PD-L1 expression on bone marrow stromal cells, respectively, after co-culturing with NKTCL cells (Figures 2, 3).

### PD-L1 Expression on Monocytes and Its Correlation With Clinical Characteristic in NKTCL Patients

**Figure 4:**
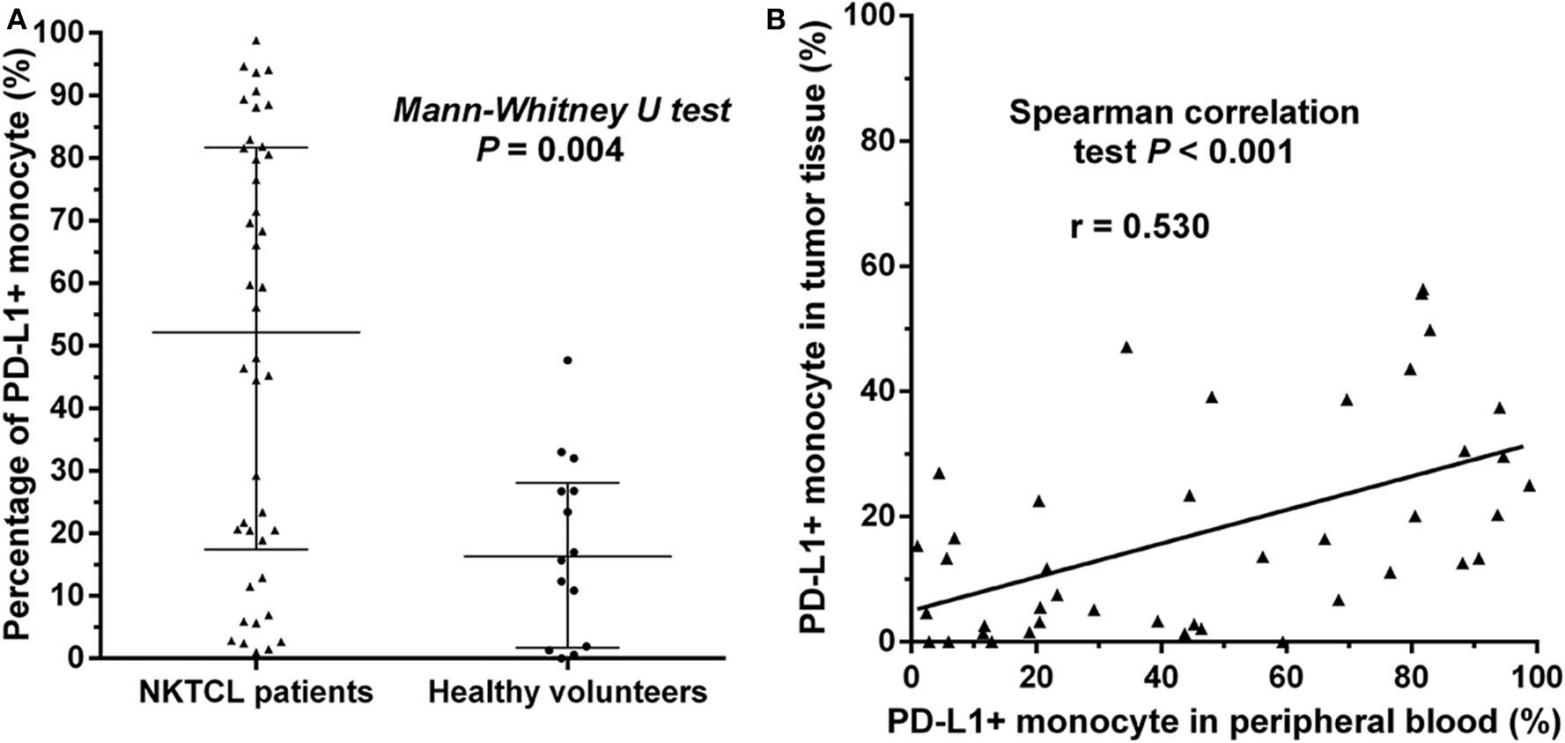
**(A)** Percentage of PD-L1+ monocytes in NKTCL patients and healthy individuals, calculated using PD-L1+ monocytes/all monocytes in peripheral blood detected by flow cytometry. The median value with interquartile range is indicated. **(B)** Correlation of percentage of PD-L1+ monocytes in peripheral blood and that in tumor tissues. The latter is calculated using PD-L1+ monocytes/all monocytes in paraffin-embedded tumor tissues examined by immunofluorescence.

We retrospectively examined the levels of PD-L1 expression on monocytes in peripheral blood and paraffin-embedded tumor tissues in 42 patients with newly diagnosed NKTCL. The percentage of PD-L1+ monocytes among all monocytes in peripheral blood was significantly higher in NKTCL patients than that in healthy individuals (Figure 4A, *P* = 0.004). Among NKTCL patients, percentage of PD-L1+ monocytes positively correlated with that in tumor tissues (Figure 4B, *P* < 0.001).

The level of PD-L1 expression on monocytes significantly correlated with some clinical characteristics in our cohort (Table 1). Patients with advanced-stage disease had significantly higher median percentage of PD-L1+ monocytes in both peripheral blood (81.8 vs. 34.4%, *P* = 0.007) and tumor tissues (39.1 vs. 11.1%, *P* < 0.001), compared with those with early-stage disease. A low-risk NKPI score (0–1) was associated with a lower percentage of PD-L1+ monocytes than a medium- to high-risk NKPI score (2–4). The median percentage of PD-L1+ monocytes was much higher in patients who experienced disease progression than that in patients without progression (81.8 vs. 23.4% in blood, *P* < 0.001; 39.1 vs. 6.7% in tumor, *P* < 0.001). No significant association was observed between PD-L1 expression on monocytes and gender, age, B symptoms, LDH level, or treatment response.

### PD-L1 Expression on Monocytes Correlated With Prognosis in NKTCL Patients

**Figure 5:**
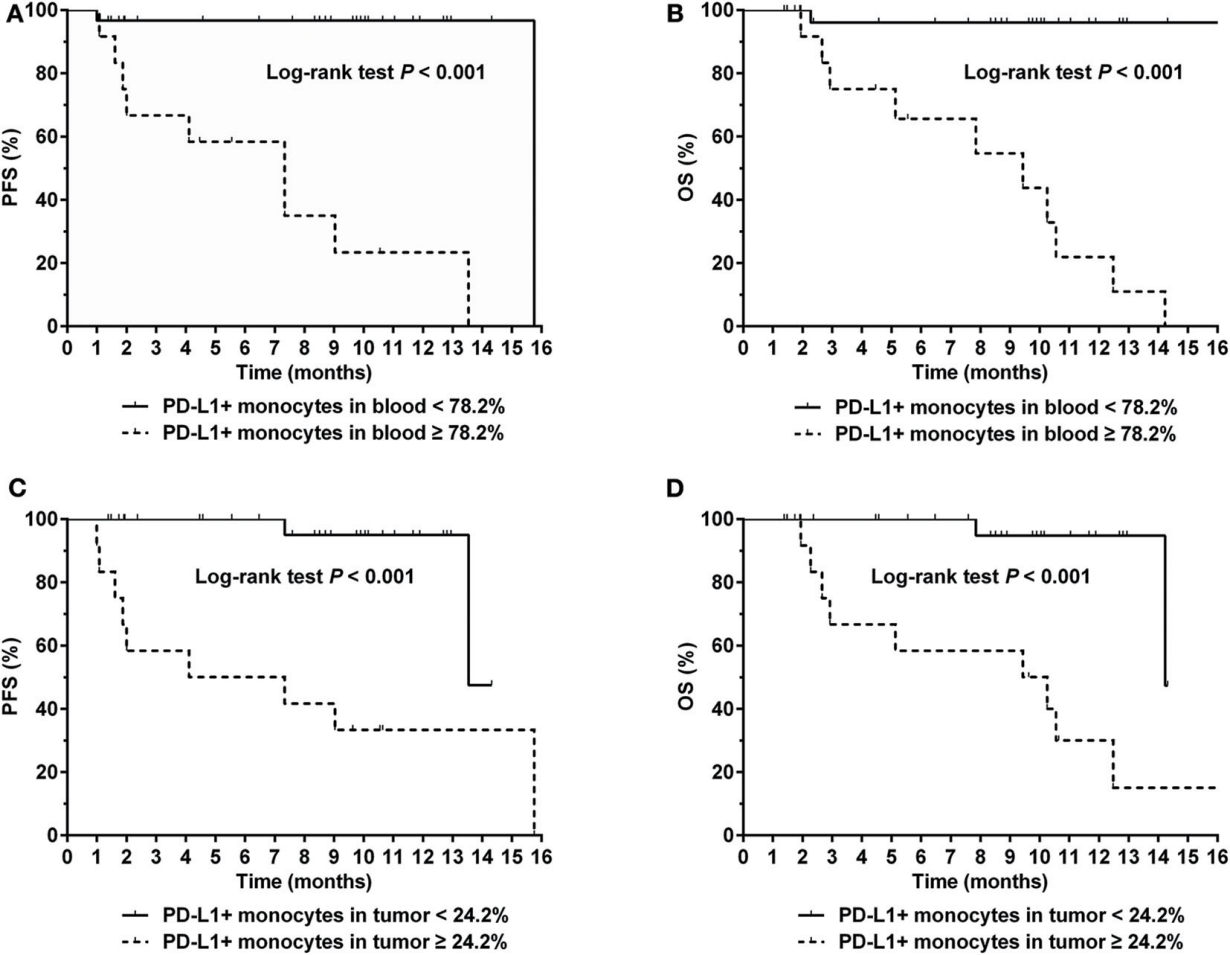
**(A)** Progression-free survival (PFS) and **(B)** Overall survival (OS) for NKTCL patients with a percentage of PD-L1+ monocytes in blood < 78.2 and ≥ 78.2%, respectively. **(C)** PFS and **(D)** OS for NKTCL patients with a percentage of PD-L1+ monocytes in tumor tissue < 24.2 and ≥ 24.2%, respectively.

The optimal cut-off value for percentage of PD-L1+ monocytes in blood to predict disease progression was 78.2% based on the ROC analysis. Patients with a higher percentage (≥78.2%) of PD-L1+ monocytes in blood had a significantly inferior survival compared with those with a lower percentage (<78.2%) of PD-L1+ monocytes in blood (median PFS: 7.3 vs. 15.7 months, *P* < 0.001, Figure 5A; median OS: 9.4 months vs. unreached, *P* < 0.001, Figure 5B). In the Cox regression model, a higher percentage (≥78.2%) of PD-L1+ monocytes in blood remained an independent adverse prognostic factor for PFS and OS (Table 2).

**Table 2:** Univariate and multivariate analysis of prognostic factors in patients with NK/T-cell lymphoma (Model 1).

**Variable**	**Progression-free survival**			**Overall survival**		
	**Univariate analysis**	**Multivariate analysis**		**Univariate analysis**	**Multivariate analysis**	
	***P-*value**	**HR (95% CI)**	***P-*value**	***P-*value**	**HR (95% CI)**	***P-*value**
Gender (female vs. male)	0.485			0.631		
Age (≥ 60 vs. < 60 years)	0.604			0.801		
ECOG score (≥ 2 vs. 0–1)	0.001			0.002		
Stage (III–IV vs. I–II)	0.003			0.002		
B symptoms (yes vs. no)	0.112			0.103		
LDH (elevated vs. normal)	0.817			0.251		
NKPI (2–4 vs. 0–1)	0.003			0.003		
Treatment response (non-CR vs. CR)	0.009			0.014		
PD-L1+ monocytes in blood (≥ 78.2 vs. < 78.2%)	0.002	11.5 (1.3–103.4)	0.029	0.002	10.8 (1.3–93.1)	0.031

*CI, confidence interval; CR, complete remission; ECOG, Eastern Cooperative Oncology Group; HR, hazard ratio; LDH, lactate dehydrogenase; NKPI, natural killer/T-cell lymphoma prognostic index*.

**Figure 6:**
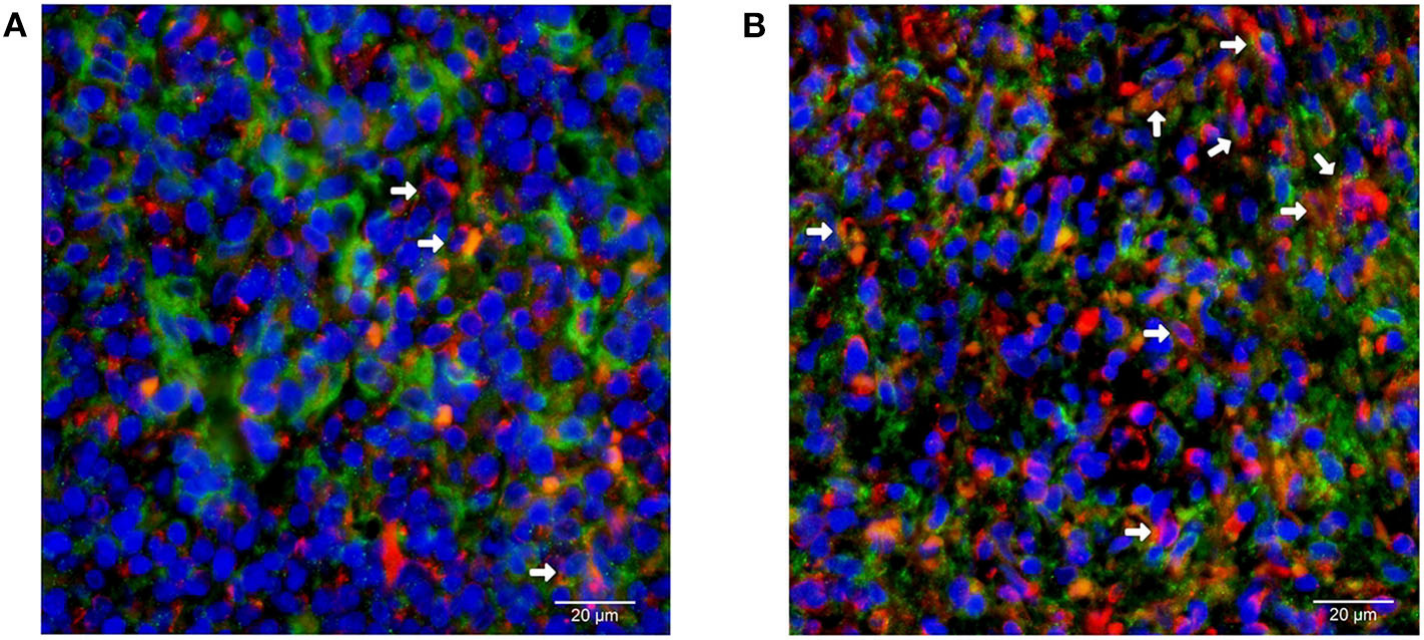
Immunofluorescent double staining of CD14 and PD-L1 in tumor tissues from patients with natural killer/T-cell lymphoma. Representative images of **(A)** low and **(B)** high percentage of monocytes with positive membrane staining of PD-L1 (red) among all CD14 (green)-positive monocytes are shown (400 × magnification).

Representative images of immunofluorescent CD14 and PD-L1 double staining in tumor tissues are shown in Figure 6. The optimal cut-off value for percentage of PD-L1+ monocytes in tumor tissues to predict disease progression was 24.2% based on the ROC analysis. Univariate analysis showed a significantly worse survival in patients with a higher percentage (≥24.2%) of PD-L1+ monocytes in tumor tissues compared with their counterparts (median PFS: 5.7 vs. 13.5 months, *P* < 0.001, Figure 5C; median OS: 9.8 vs. 14.2 months, *P* < 0.001, Figure 5D). Multivariate analysis found a higher percentage (≥24.2%) of PD-L1+ monocytes in tumor tissue an independent adverse prognostic factor for PFS but not for OS (Table 3).

**Table 3:** Univariate and multivariate analysis of prognostic factors in patients with NK/T-cell lymphoma (Model 2).

**Variable**	**Progression-free survival**			**Overall survival**		
	**Univariate analysis**	**Multivariate analysis**		**Univariate analysis**	**Multivariate analysis**	
	***P-*value**	**HR (95% CI)**	***P-*value**	***P-*value**	**HR (95% CI)**	***P-*value**
Gender (female vs. male)	0.485			0.631		
Age (≥ 60 vs. < 60 years)	0.604			0.801		
ECOG score (≥ 2 vs. 0–1)	0.001			0.002		
Stage (III–IV vs. I–II)	0.003			0.002		
B symptoms (yes vs. no)	0.112			0.103		
LDH (elevated vs. normal)	0.817			0.251		
NKPI (2–4 vs. 0–1)	0.003	14.3 (1.2–165.0)	0.033	0.003	24.2 (3.1–191.5)	0.003
Treatment response (non-CR vs. CR)	0.009	36.4 (3.1–428.7)	0.004	0.014		
PD-L1+ monocytes in tumor tissue (≥ 24.2 vs. < 24.2%)	0.002	24.1 (2.2–258.6)	0.009	0.003		

*CI, confidence interval; CR, complete remission; ECOG, Eastern Cooperative Oncology Group; HR, hazard ratio; LDH, lactate dehydrogenase; NKPI, natural killer/T-cell lymphoma prognostic index*.

## Discussion

In the present study, we discovered that NKTCL cells up-regulated expression of PD-L1 on tumor-infiltrating stromal cells, especially in monocytes, at both *in vitro* and *in vivo* levels. We also found a high level of PD-L1 expression on monocytes independently predicted poor prognosis in NKTCL patients. Taken together, expression of PD-L1 on monocytes in NKTCL patients could be an important biomarker for clinical treatment and prognosis.

PD-1/PD-L1 is a novel target for immune therapy. Previous studies have reported patients who benefit from PD-1 blockade therapy (20, 32). Several studies also pointed out immune therapy may be a promising therapeutic strategy for NKTCL (17, 33). The clinical treatment efficacy for NKTCL may be improved by better understanding the immune escape mechanism in NKTCL, and by identifying more suitable immune therapeutic markers.

Generally speaking, tumor cells evade from immune surveillance by expressing high levels of PD-L1 on themselves (12, 34). However, researchers have discovered that expression of PD-L1 on tumor-infiltrating non-malignant cells could also be elevated (35–37). The mechanism of how tumor cells interact with tumor-infiltrating non-malignant cells and elevate the PD-L1 expression on TME cells in NKTCL has not been reported yet. In our study, we reported for the first time that NKTCL cells significantly up-regulated expression of PD-L1 on tumor-infiltrating stromal cells, and this unique interaction between NKTCL cells and tumor-infiltrating stromal cells could be a novel mechanism for immune evasion of NKTCL.

Expression of PD-1 or PD-L1 has been proved as an important prognostic factor for multiple neoplasms, as well as an indicator for treatment (13, 16–18). In our previous study, we have demonstrated that a high level of PD-L1 on NKTCL cells correlates with a worse prognosis (18). With development of TME research, it has reached a consensus that PD-L1 expression on TME cells can also be a biomarker for the efficacy of immune therapy (38). Ishii has discovered that infiltrating monocytes enhance the growth of tumor cells by cell contact-dependent interaction (29). In our study, we observed NKTCL cells up-regulated the expression of PD-L1 on monocytes by cell contact interaction. We also found out the percentage of PD-L1+ monocytes in tumor tissues served as an independent prognostic factor for PFS and OS. Furthermore, the percentage of PD-L1+ monocytes in peripheral blood positively correlated with that in tumor tissues, which also remained a clinical prognostic factor with relatively easy access. In addition, patients with a low percentage of PD-L1+ monocytes in blood and tumor tissues had excellent clinical prognosis in our cohort (Figure 5). Whereas, patients with a high percentage of PD-L1+ monocytes reached for very dismal prognosis, suggesting that those patients could be more likely benefit from immune therapy. Taken together, our results indicated that PD-L1 expression on monocytes could serve as an optimal therapeutic indicator for anti-PD-L1 immune therapy for NKTCL patients, which needed further validation in prospective clinical cohorts.

Several limitations existed in this retrospective study. This was a study using a small cohort of NKTCL patients with short-term follow-up. Expressions of PD-L1 on other types of tumor-infiltrating stomal cells and their relationships with clinical prognosis were not analyzed. The mechanism of how NKTCL cells up-regulated the PD-L1 expression on monocytes remained to be further discovered and validated in future studies. Additionally, there were only four cases with primary extranasal disease in our cohort, and the findings of our study require further validation in patients with non-nasal NKTCL.

In conclusion, PD-L1 expression on monocytes is an important prognostic factor in patients with NKTCL. Whether it could serve as a biomarker to predict efficacy of anti-PD-L1 therapy worth further investigation.

## Data Availability Statement

The authenticity of this article has been validated by uploading the key raw data onto the Research Data Deposit public platform (www.researchdata.org.cn), with the approval RDD number as RDDB2020000811.

## Ethics Statement

The studies involving human participants were reviewed and approved by the Ethics Committee of Sun Yat-sen University Cancer Center. The patients/participants provided their written informed consent to participate in this study.

## Author Contributions

XZ, XB, and PL performed the *in vitro* experiments. XZ, XB, PL, ZL, MN, HY, and DL collected patients' information and samples. XB, XZ, and PL analyzed the data and wrote the manuscript. YX, WJ, and WZ designed and supervised the research. All authors read and approved the final manuscript.

## Conflict of Interest

The authors declare that the research was conducted in the absence of any commercial or financial relationships that could be construed as a potential conflict of interest.
